# Dual STAT-3 and IL-6R inhibition with stattic and tocilizumab decreases migration, invasion and proliferation of prostate cancer cells by targeting the IL-6/IL-6R/STAT-3 axis

**DOI:** 10.3892/or.2022.8349

**Published:** 2022-06-14

**Authors:** Anibal Méndez-Clemente, Alejandro Bravo-Cuellar, Salvador González-Ochoa, Maria Santiago-Mercado, Luis Palafox-Mariscal, Luis Jave-Suárez, Fabiola Solorzano-Ibarra, Maria Villaseñor-García, Pablo Ortiz-Lazareno, Georgina Hernández-Flores

**Affiliations:** 1 Doctoral Program in Biomedical Sciences Orientation Immunology, University Center for Health Sciences (CUCS), University of Guadalajara (UdeG), Guadalajara, Jalisco 44340, México; 2 Immunology Division, Western Biomedical Research Center, Mexican Social Security Institute, Guadalajara, Jalisco 44340, México; 3 Department of Health Sciences, Los Altos University Center (CUAltos), University of Guadalajara (UdeG), Tepatitlán de Morelos, Jalisco 47620, México; 4 Chronic Degenerative Diseases Research Institute Postdoctoral Stays Program for Mexico 2021, Department of Molecular and Genomic Biology, University of Guadalajara (UdeG), University Center for Health Sciences (CUCS), Guadalajara, Jalisco 44340, México; 5 Pharmacobiology Department, University Center for Exact Sciences and Engineering, University of Guadalajara (UdeG), Guadalajara, Jalisco 44340, México

**Keywords:** prostate cancer, STAT-3, IL-6 receptor, clonogenicity, migration, invasion, tocilizumab, stattic

## Abstract

Prostate cancer (PCa) is a key public health problem worldwide; at diagnosis, a high percentage of patients exhibit tumor cell invasion of adjacent tissue. STAT-3, IL-6 receptor (R) and IL-6 serum levels are associated with enhanced PCa migratory, invasive, clonogenic and metastatic ability. Inhibiting the STAT-3 pathway at different levels (cytokines, receptors, and kinases) exhibits relative success in cancer. The present study investigated the effect of Stattic (Stt) + Tocilizumab (Tcz) on proliferative, clonogenic, migratory and invasive ability of human metastatic PCa (assessed by colony formation, wound healing and migration assay). RWPE-1 (epithelial prostate immortalized cells), 22Rv1 (Tumor cells), LNCaP (Metastatic cells) and DU-145 (metastatic, castration-resistant prostate cells) cells were used *in vitro* to evaluate levels of cytokines, chemokines, growth factors (Cytometric Bead Array), STAT-3, phosphorylated STAT-3 (In-Cell Western), IL-6R, vimentin and epithelial (E-) cadherin (Western Blot). The effect of inhibition of STAT-3 (expressed constitutively in DU-145 cells) with Stt and/or Tcz on expression levels of vimentin, VEGF, and E-cadherin, as well as proliferative, clonogenic, migratory and invasive capacity of metastatic PCa cells was assessed. The expression levels of IL-6, C-X-C chemokine ligand 8, VEGF and vimentin, as well as proliferation and migration, were increased in metastatic PCa cells. Treatment with Stt or Tcz decreased vimentin and VEGF and increased E-cadherin expression levels and inhibited proliferative, clonogenic, migratory and invasive capacity of DU-145 cells; addition of IL-6 decreased this inhibitory effect. However, Stt + Tcz maintained inhibition even in the present of high concentrations of IL-6. Stt + Tcz decreased expression of vimentin and VEGF and inhibited the proliferative, clonogenic, migratory and invasive capacity of metastatic PCa cells. To the best of our knowledge, the present study is the first to combine Stt, a STAT-3 inhibitor, with Tcz, an antibody against IL-6R, to target tumor cells.

## Introduction

Prostate cancer (PCa) is the second most prevalent neoplasm in males. In 2020, it represented 14.1% of all oncological disease globally and was responsible for 6.8% of cancer-associated mortality in males (1). The presence of proinflammatory cytokines in the tumor microenvironment serves a key role in regulating cellular events in cancer including cell transformation, malignancy and migration (2,3). IL-6 has been proposed as a predictor of malignancy in multiple types of cancer where it is associated with tumor progression and decreased overall patient survival (2–4). Cytokines function as a growth factor and increase the metastatic capacity of cells via induction of tumor angiogenesis and adhesiveness (5). The high heterogeneity of cancer, even in the same tumor niche, confers different clinical manifestation, secretory capacity, and survival (6).

The biological activity of IL-6 is initiated when the cytokine binds to its receptor (IL-6R). Dimerization of IL-6/IL-6R is a key activator of Janus kinase (JAK)/STAT-3 signaling via phosphorylated (p)STAT-3, resulting in a dimeric form of STAT-3 and subsequent translocation into the nucleus by importins (7). In the nucleus, STAT-3 promotes cell proliferation and survival via downregulation of Bcl-XL, and, p53, upregulation of Mcl, and cMyc; increases invasive capacity by upregulation of matrix metalloproteinase (MMP)-2, MMP-9, MMP-7 and vimentin; cell migration via upregulation of Rho and Rac and angiogenesis by the upregulation of VEGF and hypoxia-inducible factor α (8), a marker of JAK/STAT-3 activation (9). In PCa, pSTAT-3 has been found in 95% of metastatic niches (10). STAT-3 activation is associated with increased migratory and invasive capacity via downregulation of E-cadherin and upregulation of vimentin and MMP-2 and 9. This increases mortality mediated by novel metastatic niches and resistance to death process via epithelial-mesenchymal transition (EMT) (11–14).

Due to the role of STAT-3 in cancer initiation and development, multiple treatments aim to inhibit the IL-6/IL-6R/STAT-3 signaling pathway, one of these treatments is tocilizumab (Tcz), a humanized monoclonal antibody that binds to membrane-bound and soluble IL-6R to inhibit IL-6 signaling (15). It is approved in United States of America by the Food and Drug Administration and is used clinically to treat rheumatoid and juvenile idiopathic arthritis (15). Tcz has an anti-proliferative effect on glioma cells by inhibiting the JAK/STAT-3 pathway (16). Tcz enhances the toxicity of cisplatin and decreases proangiogenic potential in a *in vitro* model of triple-negative breast cancer (MDA-MB-231) (17). Also, blockade of the STAT-3 pathway inhibits migratory capacity in breast cancer cells (18). The small compound stattic (Stt) binds directly to the SH_2_ domain of STAT-3 and inhibits JAK-induced phosphorylation (19). Stt increases the apoptotic rate of cancer cell lines MDA-MB-231 and MDA-MB-435S (19) and MA-891 (20) and tumor mouse models MV4-11 (21) by inhibiting STAT-3 activation and the nuclear translocation (19–21).

In PCa, overexpression and activation of STAT-3 increases cell proliferation, migration and invasion, processes that are necessary for development of metastasis (10). Therefore, the present study investigated the effect of the disruption of IL-6/IL-6R/STAT-3 signaling by Stt and Tcz on the migratory, invasive, proliferative, and clonogenic capacity of metastatic PCa cells.

## Materials and methods

### Reagents

STAT-3 inhibitor Stt was purchased from Santa Cruz Biotechnology, Inc. and used at non-toxic doses below the reported half-maximal inhibitory concentration (<10 µM) (19). Stt was dissolved in DMSO (Sigma-Aldrich; Merck KGaA) to create 50 mM stock solution and stored in aliquots at −80°C until use. The IL-6R humanized monoclonal antibody Tcz was purchased from Roche Diagnostics GmbH as Actemra/RoActemra^®^.

### Cell culture conditions

The non-tumor immortalized human prostate epithelial cell line RWPE-1 was maintained in keratinocyte serum-free medium containing 0.5 mg/ml bovine pituitary extract, 5 ng/ml human recombinant EGF supplement and penicillin/streptomycin (100 IU/ml; Gibco; Thermo Fisher Scientific, Inc.). The prostate carcinoma cell lines 22Rv-1, LNCaP and DU-145 were maintained in RPMI-1460 culture medium supplemented with 10% fetal bovine serum (FBS) and penicillin/streptomycin (100 IU/ml; all Gibco; Thermo Fisher Scientific, Inc.). All cell lines were obtained from American Type Culture Collection. All cultures were maintained at 37°C in 5% CO_2_ and routinely tested for mycoplasma; cells were negative throughout the study. When PCa cells reached 80% confluence, they were harvested with Trypsin^®^ (Gibco; Thermo Fisher Scientific, Inc.) and seeded at different densities for subsequent experiments.

### Cell treatment

DU-145 cells were treated at 37°C for 24–72 h with Stt (3 µM), Tcz (10 µg/ml) or IL-6 (50 ng/ml) or their simultaneous combinations as follows: Stt + IL-6; Tcz + IL-6; Stt + Tcz or Stt + Tcz + IL-6. For Stt + IL-6, Tcz + IL-6 and Stt + Tcz + IL-6, addition of IL-6 was performed at 1 h following treatment with Stt and/or Tcz. An untreated control group (UCG) did not receive any treatment. The cells were cultured overnight at 37°C prior to treatment to allow cells to attach to the plates.

### Supernatant of prostate-derived RWPE-1, 22Rv1, LNCaP and DU-145 cell lines

PCa 22Rv1, LNCaP, DU-145, and RWPE-1 cells were grown in 75-cm^2^ flasks to 90% confluence. Cells were harvested using Trypsin solution (Gibco; Thermo Fisher Scientific, Inc.). A total of 250,000 cells was plated into a 25-cm^2^ flask containing fresh medium. Cultures were maintained at 37°C in a humidified atmosphere with 5% CO_2_. Following 12, 24 and 48 h incubation, supernatant was collected under sterile conditions, centrifuged at 4°C, 1,400 × g for 10 min, and filtered through a membrane (0.2 µm; MilliporeSigma). The supernatant was stored at −80°C until use.

### Flow cytometry assessment of cytokines and growth factor

Bead-based multiplex assay was used to quantify cytokines and growth factor in supernatant of prostate-derived cell lines using LEGENDplex™ HU Essential Immune Response Panel and LEGENDplex Human Growth Factor Panel (cat. no. 741061 and 740929 respectively; both BioLegend, Inc.) according to the manufacturer's instructions. Briefly, 25 µl thawed supernatant, diluted standard and blanks were added to the corresponding tubes; 25 µl pre-mixed beads and detection antibodies was added to all tubes (Growth Factor Panel Detection Antibodies, cat. no, 76303 and Human Essential Immune Response Panel Antibodies Cat. no. 750000547 both BioLegend, Inc.). Tubes were incubated for 2 h at room temperature with shaking. Then, 25 µl Streptavidin-Phycoerythrin conjugate was added, tubes were incubated for 30 min at room temperature, washed and suspended in 200 µl wash buffer. Data were acquired using an Attune Acoustic Focusing Cytometer (Thermo Fisher Scientific, Inc.) and analyzed utilizing LEGENDplex version 8.0 (BioLegend, Inc.). All results are expressed in pg/ml.

### VEGF measurement by ELISA

The secreted VEGF in DU-145 cell line was determined quantitatively using a commercial ELISA kit (cat. no. DVE00, Quantikine Human VEGF; R&D Systems, Inc.) according to the manufacturer's instructions. In brief, cell supernatant was diluted at 1:3 with assay diluent, 150 µl supernatant was added to wells, incubated at room temperature for 2 h and washed three times with 300 µl wash buffer. Then, 200 µl Human VEGF Conjugate was added to each well and incubated and washed as aforementioned. Finally, 200 µl substrate solution was added, color was developed for 20 min and the reaction was stopped with 50 µl STOP solution. Absorbance was measured at 450 nm using a microplate reader (Synergy HT; BioTek Instruments, Inc.); data are expressed as pg/ml (analyzed using Gene5, version 3.08, biotek.com/products/software-robotics–software/gen5-microplate-reader–and-imager-software/).

### WST-1 cell viability assay

PCa cells were seeded at a density of 2×10^5^ cells/well in a 96-well plate and cultured overnight at 37°C to allow attachment. The medium was replaced with fresh medium containing Stt (1, 3, 5, 7 or 10 µM) or Tcz (10, 100, 1,000 or 10,000 ng/ml) for 24 h at 37°C, Etoposide at 5 µM was used as a positive control Then, 10 µl WST-1 reagent solution (Sigma-Aldrich; Merck KGaA) was added for 2 h to each well. Absorbance was read at 480 nm with a reference wavelength of 650 nm using a microplate reader (Synergy HT; BioTek Instruments, Inc.). A total of three independent experiments were performed in triplicate. All readings were normalized to the UCG.

### Real-time cell proliferation monitoring

xCELLigence DP (Roche Diagnostics GmbH) was used to monitor cell proliferation in real time. This system allows the measurement of impedance in real time; the higher the impedance value, the higher the number of live cells growing on the surface of each well; a maximal value is reached when the cell layer becomes confluent (22). Treated or untreated RWPE-1, 22Rv1, LNCaP, and DU-145 cells were seeded at a density of 5×10^3^ cells/well in quadruplicate in electronic microtiter plates (E-Plate; Roche Diagnostics GmbH) and measured with programmed signal detection every 20 min for 92 h with the xCELLigence system according to the manufacturer's instructions. Data acquisition and analysis were performed with RTCA software (version 1.2; Roche Diagnostics GmbH).

### Wound-healing assay

Scratch wound-healing assay was performed to determine cell migration using confluent cultures (80–90% confluence). Briefly, 1×10^5^ cells/ml were seeded in a 6-well tissue culture. After cells reached confluency, they were starved for 24 h using 0.2% serum in growth medium. At 2 h before wound healing assay cells were treated with mitomycin (5 µg/ml) at 37°C for 2 h to inhibit cell proliferation, as previously described (23). A sterile p200 pipette tip was used to create a wound on the confluent monolayer and culture medium was replenished. Images of the scratch area were recorded using a guide-dot previously drawn underneath the plates at 0, 6, 12 and 24 h. The experiments were repeated three times in triplicate. Images were captured using AxioCam ERc5s inverted phase-contrast microscope (Primo Vert, Cat. no. 415510-1101-000; Zeiss AG) at 40× magnification. ImageJ software (version 1.8.0_172; National Institutes of Health) was used to calculate wound area. Each group was compared with the UCG. Closure rate (%) was calculated as follows: (Initial wound width-final wound width)/initial wound width ×100.

### Matrigel invasion assay

Invasion capacity was determined in DU-145 cells via chemotaxis assay. Transwell chambers with 8-µm pore polycarbonate filters (Wuxi NEST Biotechnology Co., Ltd.) were coated with Matrigel diluted in RPMI serum-free medium at a final concentration of 0.25 mg/ml for 24 h at 37°C prior the beginning of the invasion assay.

Briefly, DU-145 cells were seeded at 5×10^5^ cells/ml in 6-well plates and cultured at 37°C in RPMI Supplemented medium (10% FBS and penicillin/streptomycin 100 IU/ml) for 24 h. Then, the cells were washed with PBS and cultured for another 72 h at 37°C with Stt, Tcz and/or IL-6. The medium was replaced 24 h before the beginning of the invasion assay with RPMI serum-free medium (Gibco; Thermo Fisher Scientific, Inc.) with Stt, Tcz and/or IL-6 at 37°C.

For the assay, cells were harvested with Trypsin and resuspended in RPMI serum-free medium with Stt, Tcz and/or IL-6 at 5×10^5^ cell/ml, then 150 µl cell suspension was added to a Transwell chamber. RPMI culture medium with 10% FBS (both Gibco; Thermo Fisher Scientific, Inc.) used as a chemoattractant was added to the lower chambers and cells were allowed to invade through a porous membrane coated with Matrigel and incubated at 37°C for 48 h. Invading cells on the membrane were fixed in 3.7% paraformaldehyde at room temperature for 15 min and stained for 30 min at room temperature with Sulforhodamine B (SRB) (0.057% wt/vol). A total of five fields of view were observed using a Zeiss Primo Vert microscope light microscope at 100× magnification and photographed with AxioCam ERc5s.

SRB that became attached to cells was recovered and measured as previously described by Vichai and Kirtikara (24). Briefly, the Transwell inserts were placed on a new p24 plate with 500 µl 10 mM Tris base (pH, 10.5) for 30 min at room temperature. Aliquots of 100 µl recovered SRB were transferred onto a 96-well plate and the optical intensity was measured at 510 nm using a microplate reader. Invasion (%) was normalized to the control as follows: (Absorbance in treatment group/absorbance in UCG) ×100.

### Colony number formation assay

DU-145 cells were harvested at exponential growth phase, seeded at 200 cells/well in 6-well plates and cultured at 37°C for 24 h before addition of Stt, Tcz and/or IL-6 for 72 h at 37°C. Following treatment, cells were harvested, and 300 cells/well were cultured in a new 6-well plate. The cells were incubated with RPMI supplemented medium for 24 h at 37°C, then medium was replaced with fresh medium containing Stt, Tcz and/or IL-6. The cells were then incubated at 37°C for 14 days. During this time, the culture medium was replaced every 3 days with fresh supplemented medium with Stt, Tcz and/or IL-6. Cells were fixed using 3.7% formaldehyde at room temperature for 15 min and stained for 30 min at room temperature with Sulforhodamine B (SRB) (0.057% wt/vol). The colonies (>50 cells) were viewed with Zeiss Primo Vert light microscope at 40X magnification to observe the cell number in the colonies and photographed with AxioCam ERc5s. Colonies was counted with the soft ImageJ software (version 1.8.0_172; National Institutes of Health).

### Western blot analysis

Briefly, DU-145 cells were seeded at 4×10^6^ cells/ml in 150-mm plates and cultured at 37°C in RPMI Supplemented medium (10% FBS and penicillin/streptomycin 100 IU/ml) for 24 h. Then, the cells were washed with PBS and cultured for 120 h at 37°C with Stt, Tcz and/or IL-6. Untreated cells were seeded at 4×10^6^ cells/ml in 150 mm plates and cultured at 37°C in RPMI Supplemented medium (10% FBS and penicillin/streptomycin 100 IU/ml) for 24 h, and harvested at 90% of confluency.

For the protein extraction, cells were detached with a cell scraper and centrifuged at 4°C, 1,400 × g for 10 min. A total of 300 µl RIPA buffer (0.5% deoxycholate, 0.5% NP-40, 0.5% SDS, 50 mM Tris, pH 8.0 and 150 mM NaCl) with Protease Inhibitor Cocktail (Roche Applied Science) was added and cell suspensions were incubated on ice for 30 min. The lysate was sonicated at 20 kHz for 5 min at high intensity with 30 sec rest intervals with the Bioruptor Sonicator (Diagenode SA). Protein extract was obtained following 30 min incubation at 4°C and 12 min centrifugation at 13,300 × g and 4°C. Bradford Assay kit (HGHiring; Bio-Rad Laboratories, Inc.) was used to determine concentration of proteins in the samples. Samples containing 50 µg total protein were resolved via 10% SDS-PAGE. The proteins were transferred onto a PVDF membrane (0.2-µm pore) and blocked with LI-COR Odyssey Blocking Solution Ready to use (cat. no. 927-70001; LI-COR Biosciences) under agitation for 2 h at room temperature. Primary antibodies for STAT-3 (1:1,000; clone no. 124H6) and pSTAT-3 (1:2,000; clone no. M9C6) were acquired from Cell Signaling Technology, Inc.; antibodies for vimentin (1:2,000; clone no. V9), E-cadherin (1:2,000; clone no. G-10) and IL-6R (1:1,000; clone no. H-7) were obtained from Santa Cruz Biotechnology, Inc. Primary antibodies were incubated overnight at 4°C under agitation in the dark. The membranes were washed and probed with IRDye^®^ 800 secondary antibodies (1:15,000; Cat. no. 926-68028; LI-COR Biosciences) for 2 h at room temperature. Washed membranes were scanned with Odyssey™ Infrared Imaging System (LI-COR Biotechnology). Optical density was measured using Image Studio Lite ver. 5.2.5 software and normalized to β-actin coupled with Alexa Fluor^®^ 680 (1:2,000; clone no. C4; cat. no. sc-47778 AF680; Santa Cruz Biotechnology, Inc.) or with Revert 700 Total Protein Stain (TPS; cat. no. 926-11010; LI-COR Biosciences) according to manufacturer instructions. Briefly, the membranes were submerged in TPS solution for 5 min, washed twice for 30 sec at room temperature with wash solution (LI-COR Biosciences) and imaged. Membranes were scanned using the 700 nm channel of the Odyssey scanner. TPS stain was removed with reversal solution (LI-COR Biosciences) for 5 min at room temperature with gentle shaking. Optic density was measure with the software Image Studio Ver 4.0 (LI-COR Biosciences).

### Reverse transcription-quantitative (RT-q)PCR

For evaluation of genes involved in EMT, RNA extraction was performed using the commercial GeneJET RNA Purification kit (Thermo Fisher Scientific, Inc.). RNA was quantified using a plate reader (Synergy HT; BioTek Instruments, Inc.). Synthesis of cDNA was performed using commercial Transcriptor First Strand cDNA Synthesis kit (Roche Diagnostics GmbH) according to the manufacturer's protocol. qPCR was performed using LightCycler^®^ FastStart DNA Master PLUS SYBR Green I and LightCycler version 2.0 (both Roche Diagnostics GmbH). The qPCR was performed using initial denaturation at 95°C for 10 min followed by 45 cycles of denaturation at 95°C for 15 sec; annealing/extension is shown in the table I. The primer design and qPCR condition are shown in Table I. Data were normalized via the 2^−ΔCq^ method (25) using the *RPL32* as reference gene.

### In-Cell Western (ICW) assay

ICW assay was performed using Odyssey Imaging System (LI-COR Biotechnology). Briefly, 2×10^4^ cell/well DU-145 cells were seeded at 37°C for 24 h in a 96-well optical black wall clear bottom plate (Thermo Fisher Scientific, Inc.). When cells reached 60–70% confluence, supplemented medium with Stt, Tcz and/or IL-6 were applied for 24 h at 37°C. Cells were fixed with 100 µl/well methanol-acetone solution (3:1) for 20 min at −20°C. Next, cells were permeabilized with 0.5% Triton X-100 for 15 min at room temperature and left for 24 h at 4°C. Cells were blocked with LI-COR Odyssey Blocking Solution Ready to use (cat. no. 927-70001; LI-COR Biosciences) at room temperature for 2 h. The cells were incubated at 4°C overnight with mouse IgG against STAT-3 (1:1,000; Cat. no. 9193; Cell Signaling Technology, Inc.) and pSTAT-3 (1:400; Cat. no. 9193; Cell Signaling Technology, Inc.). Following five washes with Dulbecco's PBS (HyClone; Cytiva), cells were stained with goat anti-mouse IgG IRDye 800 antibody (1:5,000; cat. no. 926-68028; LI-COR Biosciences) at room temperature for 2 h. The microplates were scanned with the Odyssey CLx Infrared Imaging System (LI-COR Biosciences). The integrated fluorescence intensity representing the protein expression levels was acquired using software provided with the imager station (Odyssey Software version 3.0; LI-COR Biosciences). Fluorescence intensity of STAT-3 and pSTAT-3 was calculated by normalization to DRAQ5 (1:5,000; Cat. no. 4084; Cell Signaling) at room temperature for 2 h. Optical density was measure with Image Studio Ver 4.0 (Provided by LI-COR Biosciences).

### Statistical analysis

All experimental procedures were performed in triplicate and repeated ≥3 times unless otherwise specified. Data were analyzed using Shapiro-Wilk test to determine normality and one-way ANOVA followed by Tukey's post hoc test to compared groups. Data are presented as the mean ± standard deviation. Statistical analysis was performed using GraphPad Prism version 9.0 (GraphPad Software, Inc.). P<0.05 was considered to indicate a statistically significant difference. BioRender.com was used to create figures.

## Results

### High concentrations of IL-6 and VEGF are secreted by metastatic PCa cells

**Figure 1.**
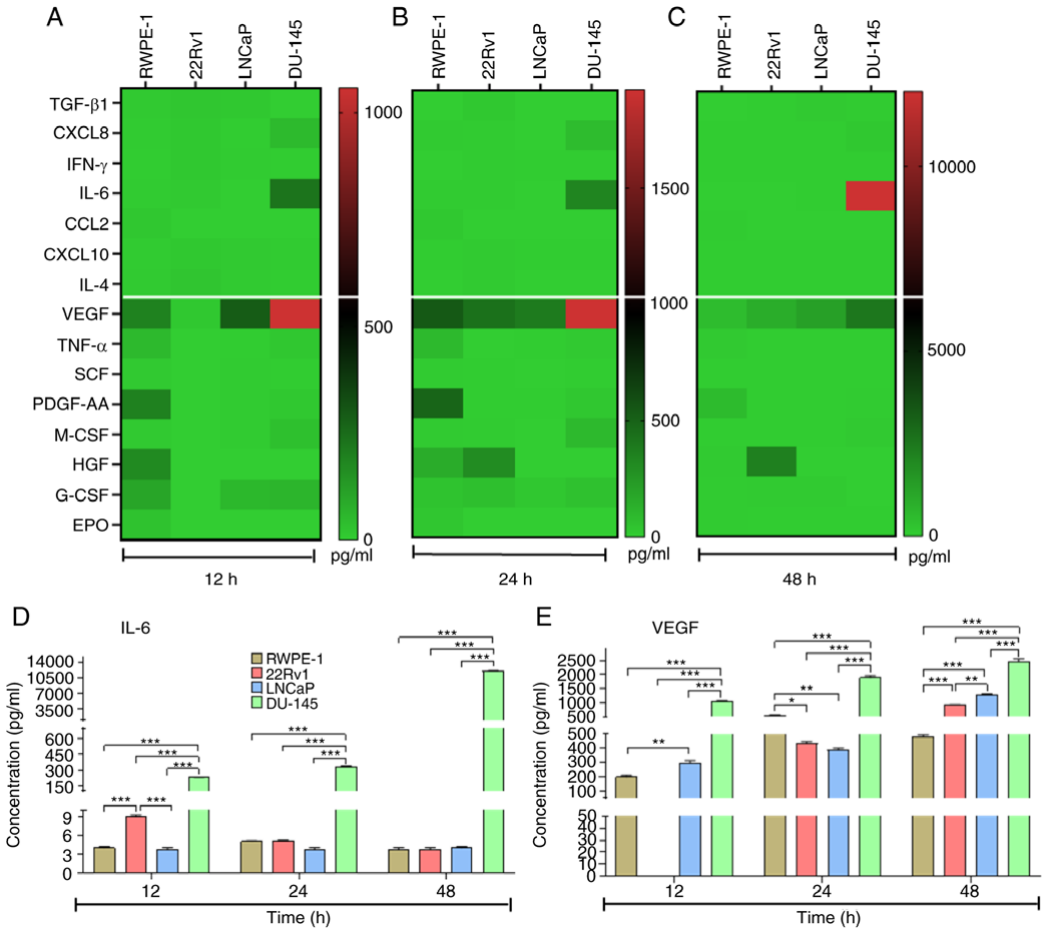
Secretion of cytokines and growth factors by prostate cells. Secretion of cytokines and growth factors in supernatant of prostate cancer (22Rv1, LNCaP and DU-145) and RWPE-1 non-tumorigenic cells at (A) 12, (B) 24 and (C) 48 h. Data are shown as heat maps. Concentration of (D) IL-6 and (E) VEGF in cell lines. Data are presented as the mean ± SD. *P<0.05, **P<0.01 and ***P<0.001. TGF, transforming growth factor; CXCL, C-X-C chemokine ligand; CCL, C-C motif chemokine ligand; VEGF, vascular endothelial growth factor; SCF, stem cell factor; PDGF, platelet-Derived Growth Factor; M-CSF, Macrophage colony-stimulating Factor; HGF, hepatocyte growth factor; G-CSF, Granulocyte-colony stimulating factor; EPO, erythropoietin.

Secretion of cytokines, chemokines was determined with CBAs assay [transforming growth factor Transforming growth factor (TGF)-β1, C-X-C chemokine ligand (CXCL)-8 (also called IL-8), INF-γ, IL-6, C-C motif chemokine ligand (CCL)-2, CXCL-10 and IL-4] and growth factors [Vascular endothelial growth factor (VEGF), Tumor necrosis factor-α, Stem cell factor, Human Platelet-Derived Growth Factor, Macrophage Colony-Stimulating Factor, hepatocyte growth factor, Granulocyte-colony stimulating factor (G-CSF) and Erythropoietin] were assessed in PCa (22Rv1, LNCaP, and DU-145) and non-tumorigenic prostate cells (RWPE-1) at 12, 24 and 48 h. PCa cells secreted high concentrations of CXCL8, IL-6, VEGF and G-CSF at 12 (Fig. 1A), 24 (Fig. 1B) and 48 h (Fig. 1C). IL-6 and VEGF secretions were higher in DU-145 than 22Rv1, LNCaP, and RWPE-1 cells (Fig. 1D and E). These results show that IL-6 and VEGF were secreted to a greater extent by metastatic cell lines. The cytokines and transcription factors expressed by cells are presented in Figs. S1 and S2.

### Expression of vimentin and pSTAT-3 is high in metastatic DU-145 cells

**Figure 2.**
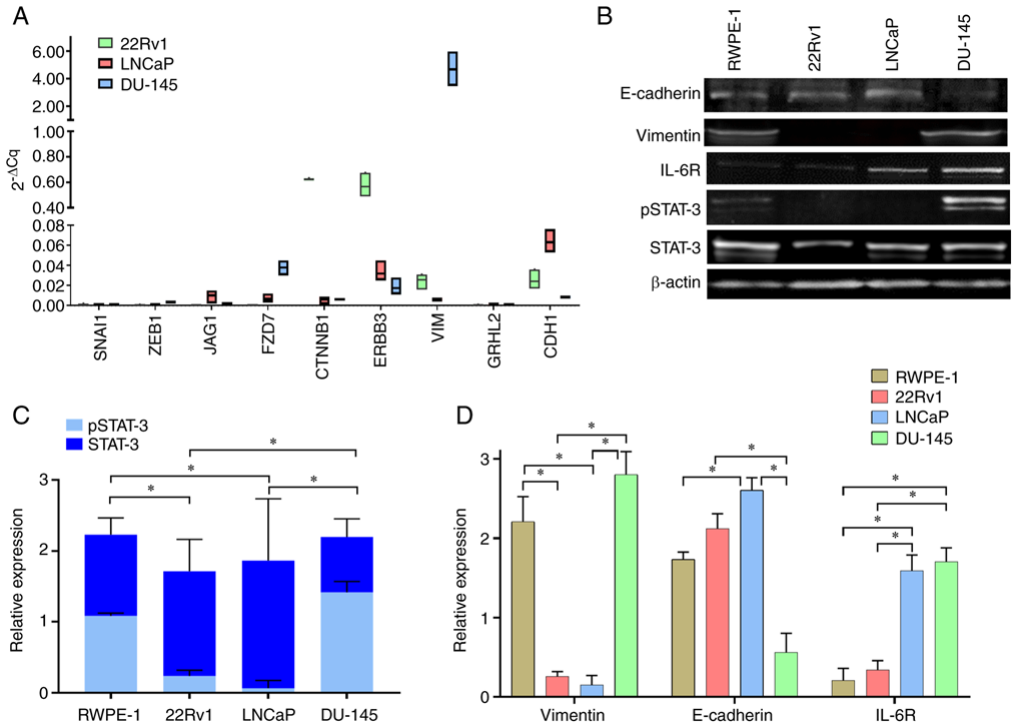
Expression of vimentin, E-cadherin, IL-6R, STAT-3 and pSTAT-3 in prostate cells. (A) Expression levels of genes associated with epithelial-mesenchymal transition (*SNAI1, ZEB1, JAG1, FZD7, CTNNB1, ERBB3, VIM, GRHL2* and *CDH1*) normalized to *RPL32* expression. (B) Protein expression levels of vimentin, E-cadherin, IL-6R and STAT-3 prostate cancer (22Rv1, LNCaP and DU-145) and RWPE-1 non-tumorigenic prostate cells was determined by western blotting. Densitometric analysis of (C) STAT-3 and pSTAT-3 and (D) vimentin, E-cadherin and IL-6R expression. Data are presented as the mean ± SD of three independent experiments. *P*<*0.05. GRHL2, grainyhead-like transcription factor 2; CDH1, epithelial-cadherin; FZD7, frizzled class receptor 7; JAG1, jagged canonical Notch ligand 1; ERBB3, Erb-b2 receptor tyrosine kinase 3; Snail1, snail family transcriptional repressor 1; R, receptor; p, phosphorylated; Zeb1, zinc finger E-box binding homeobox 1.

Expression of genes associated with EMT were assessed and an inverse association between vimentin and E-cadherin was observed; these are consistent with protein levels (Fig. 2A). DU-145 highly expressed vimentin, but LNCaP and 22Rv1 cells exhibited low expression; E-cadherin was highly expressed in LNCaP and 22Rv1 cells but its expression in DU-145 cells was low (Fig. 2A). Expression levels of E-cadherin, vimentin and other proteins associated with activation of the STAT-3 pathway (IL-6R and total and pSTAT-3) were assessed. There were different protein expression in PCa (22Rv1, LNCaP and DU-145) and RWPE-1 non-tumorigenic prostate cells (Fig. 2B). The phosphorylation was different in all cell lines: RWPE-1 and DU-145 cells exhibited increased pSTAT-3 compared with 22Rv1 and LNCaP cells (Fig. 2C). The expression of E-cadherin was lower in DU-145 compared with 22Rv1 and LNCaP cells but not significantly different compared with RWPE-1 cells; high expression of vimentin was observed in RWPE-1 and DU-145 compared with 22Rv1 and LNCaP cells. IL-6R was more highly expressed in metastatic LNCaP and DU-145 than RWPE-1 and 22Rv1 cells (Fig. 2D). The expression of vimentin was higher in cells expressing pSTAT-3; on the other hand expression of E-cadherin was lower in the same cells. These results reveal a potential association between expression of vimentin and IL-6R and downregulation of E-cadherin in a pSTAT-3- dependent matter.

### Proliferation and migration are higher in metastatic DU-145 cells

**Figure 3.**
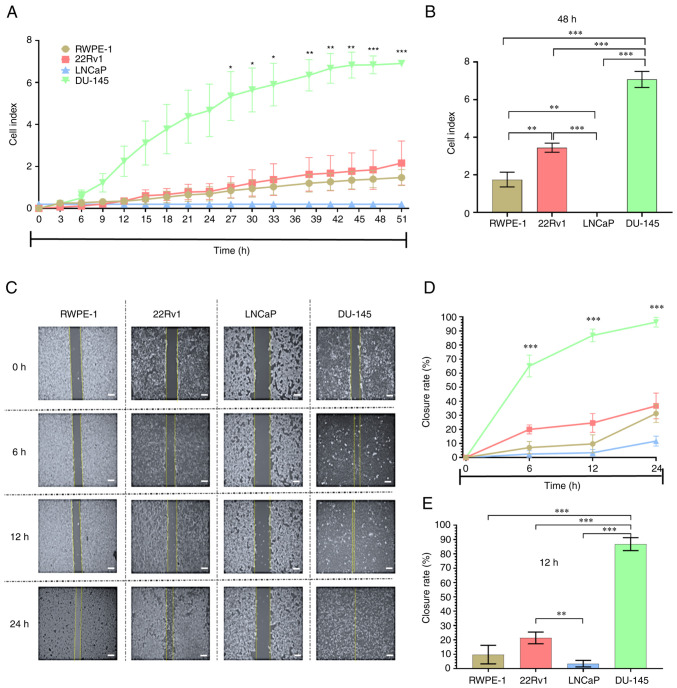
Proliferation and migration capacity of prostate cell lines (22Rv1, LNCaP and DU-145) and RWPE-1 non-tumorigenic prostate cells. (A) Proliferation analysis was performed using xCELLigence. (B) Proliferative capacity was evaluated at 48 h. (C) Wound-healing assay was used to compare migratory capacity of the cells; wound was measured at (D) 0, 6, 13 and 24 h and (E), the scale bars denote 100 µm (40× amplification), difference was determined at 12 h. Data are presented as the mean ± SD of three separate experiments. *P<0.05, **P<0.01 and ***P*<*0.001 22Rv1 vs. DU-145.

Proliferation and migration capacity were assessed in cell lines. DU-145 cells exhibited a higher proliferative capacity than other cell lines (Fig. 3A); at 48 h, proliferation was higher than in RWPE-1, 22Rv1 and LNCaP cells (Fig. 3B). Likewise, the migratory capacity of cells was different at 6, 12 and 24 h (Fig. 3C and D). The wound-closure rate was higher in DU-145 cells (Fig. 3E). The proliferative and migratory capacity exhibited a similar pattern among cell lines. The order of the proliferative and migratory capacities of the cell lines was LNCaP, RWPE-1, 22Rv1 and DU-145. These results demonstrated that the metastatic androgen-independent cell line DU-145 had higher proliferative and migratory capacity than other cell lines. IL-6 secretion and IL-6R expression results showed the role of the IL-6/pSTAT-3 circuit in DU-145 PCa cells. DU-145 cells were therefore selected to evaluate the effect of inhibition of IL-6/IL-6R/STAT-3 on the migratory, invasive, clonogenic and proliferative capacity of a cell line expressing activated STAT-3.

### Stt + Tcz decreases STAT-3 phosphorylation in DU-145 cells

**Figure 4.**
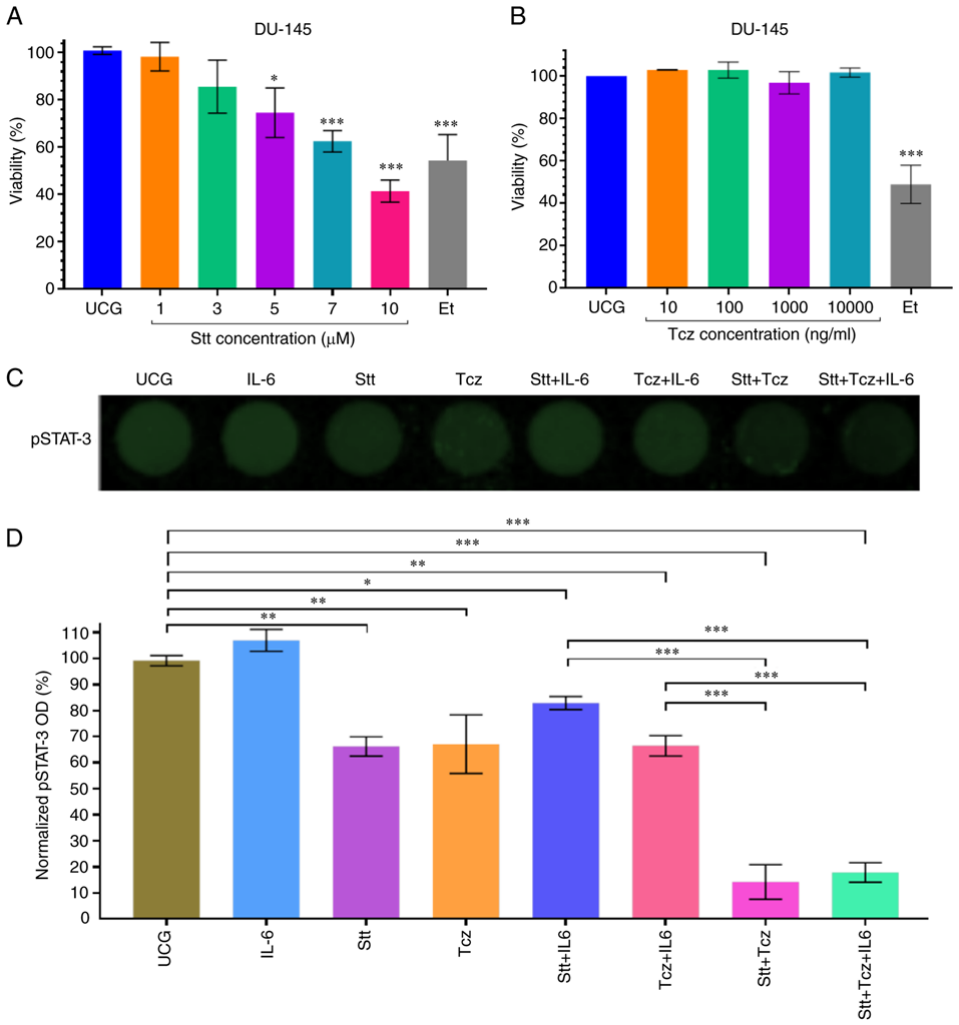
Stt + Tcz decreases pSTAT-3 at 24 h in DU-145 cells. (A) Effect of Stt on DU-145 viability was determined at 1, 3, 5, 7 and 10 µM at 24 h. (B) Effect of Tcz was determined at 10, 100, 1,000 and 10,000 ng/ml. (C) In-Cell Western assay was used to evaluate the effect of Stt, Tcz, IL-6 for 24 h. (D) Densitometric analysis. Data are presented as the mean ± SD of three independent experiments, normalized to their respective control. *P<0.05, **P<0.01 and ***P<0.001 vs. UCG. Stt, stattic; Tcz, tocilizumab; p, phosphorylated; Et, Etoposide; UCG, untreated control group; OD, optical density.

Before evaluating the effect of STAT-3 inhibition on DU-145 cells, the effect of Stt and Tcz on cell viability was assessed. Stt decreased viability in DU-145 cells at a concentration of ≥5 µM (Fig. 4A). Moreover, Tcz (10–1,000 ng/ml) did not decrease viability of DU-145 cells (Fig. 4B). Therefore, Stt at 3 µM and Tcz 10 µg/ml were selected, based on a previous report (26). The effect of Stt and/or Tcz on STAT-3 phosphorylation was assessed. Both Stt and Tcz decreased phosphorylation of STAT-3 by 40% compared with UCG (Fig. 4C and D). Following addition of IL-6, the inhibitory effect was decreased for both treatments. However, Stt + Tcz maintained high and effective inhibition. At 24 h, STAT-3 phosphorylation levels were lower than 20% even when IL-6 was added. These results indicated that IL-6 decreased the inhibitory effect produced by Stt. However, combined Stt + Tcz was more effective in inhibiting pSTAT-3 and addition of IL-6 did not significantly increase pSTAT-3 expression.

### Stt + Tcz decreases the effect of IL-6 on vimentin, E-cadherin and VEGF

**Figure 5.**
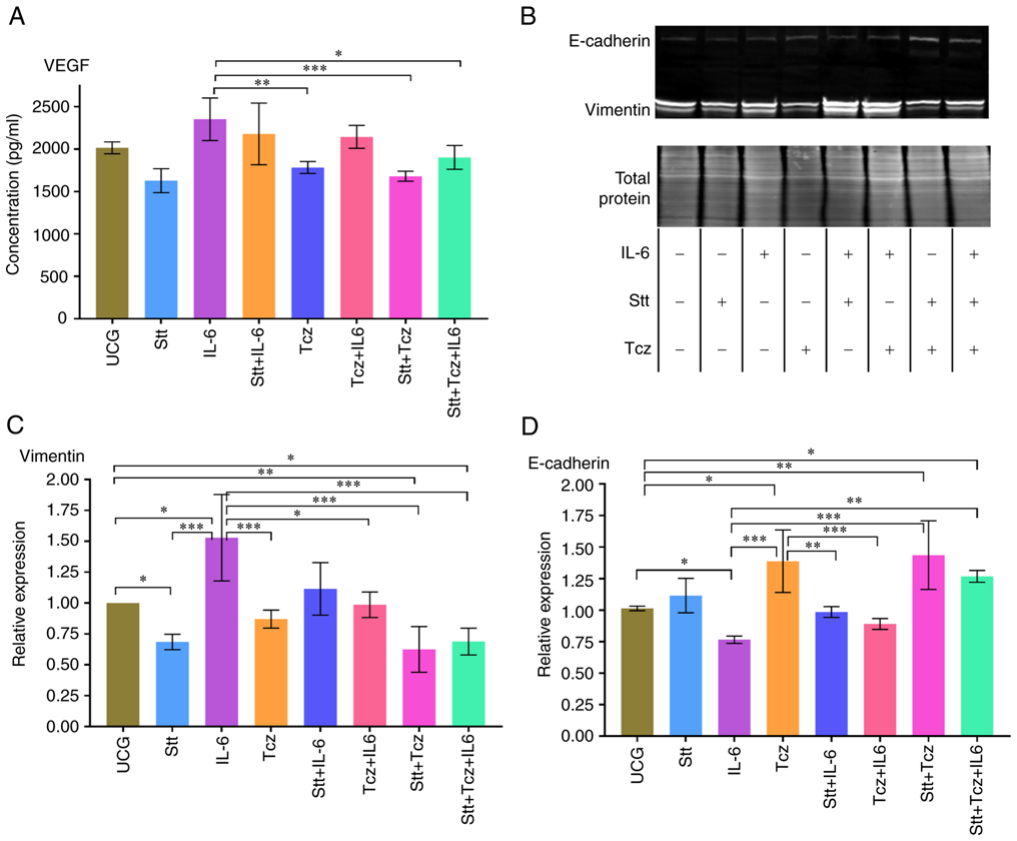
Stt + Tcz decreases VEGF and vimentin and increases E-cadherin expression induced by IL-6 in DU-145 cells. (A) VEGF in DU-145 cell supernatant was assessed by ELISA. (B) Western blotting was utilized to evaluate the differences in E-cadherin and vimentin expression. Densitometric analysis of (C) vimentin and (D) E-cadherin. Data are presented as the mean ± SD of three independent experiments. *P<0.05, **P<0.01 and ***P*<*0.001. Stt, stattic; Tcz, tocilizumab.

The effect of Stt and/or Tcz, on expression of VEGF, vimentin and E-cadherin was assessed. Both Stt and Tcz alone decreased VEGF compared with the IL-6 group in DU-145 cells. Following added IL-6 to Stt or Tcz, we did not observe this effect; however, Stt + Tcz combination decreased VEGF secretion compared with the IL-6 group (Fig. 5A). A similar effect was observed for vimentin (Fig. 5B and C); Stt + Tcz significantly decreased baseline vimentin expression in DU-145 cells and reversed IL-6-mediated induction of vimentin expression. E-cadherin, (a key molecule that maintains cell-cell adherence and regulates the Wnt pathway (27), was increased by Tcz but decreased in the IL-6 group. In the Stt + Tcz group, E-cadherin expression was increased even when IL-6 was added (Fig. 5D). These results demonstrated that combined Stt + Tcz was effective in inhibiting the effect of IL-6 on tumor cells, resulting in upregulation of E-cadherin and downregulation of vimentin and VEGF in DU-145 PCa cells.

### Stt + Tcz decreases proliferative and clonogenic capacity

**Figure 6.**
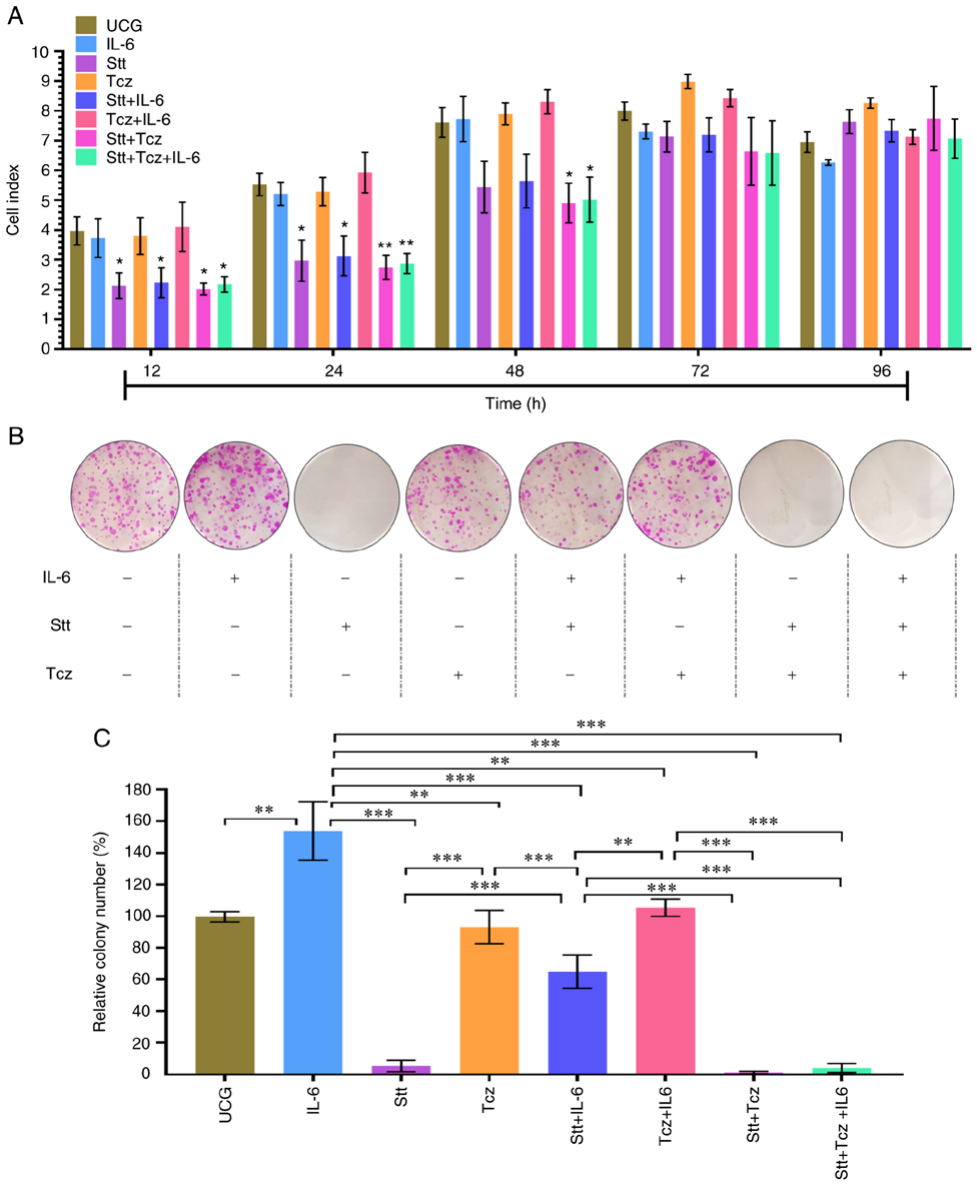
Stt + Tcz treatment decreases proliferation and clonogenic capacity of DU-145 cells. (A) Proliferation assay of DU-145 cells by xCELLigence. (B) Clonogenicity assay was performed for 14 days. (C) Cells were treated according to the scheduled treatment for 3 days after cells were detached and seeded in a 6-well plate with supplemented medium plus treatment. Data are presented as the mean ± SD of three independent experiments, normalized to control group. *P<0.05, **P<0.01 and ***P<0.001. Stt, stattic; Tcz, tocilizumab; UCG, untreated control group.

Next, proliferative capacity was assessed using xCELLigence. All groups treated with Stt exhibited decreased proliferation in a time-dependent manner from 12 to 48 h; at 72 h the effect was non-significant in comparison with UCG (Fig. 6A).

IL-6 treatment increased colony formation compared with UCG (160 vs. 100%, respectively; Fig. 6B). Stt decreased colony formation to <10% (Fig. 6C) but Stt + IL-6 treatment increased colony formation. Stt + Tcz decreased the number of colonies to <10% in comparison with UCG and Stt + Tcz + IL-6 did not increase the number of colonies significantly. This showed that Stt + Tcz was effective in decreasing colony formation capacity in the presence or absence of IL-6.

### Stt + Tcz decrease migratory and invasive capacity

**Figure 7.**
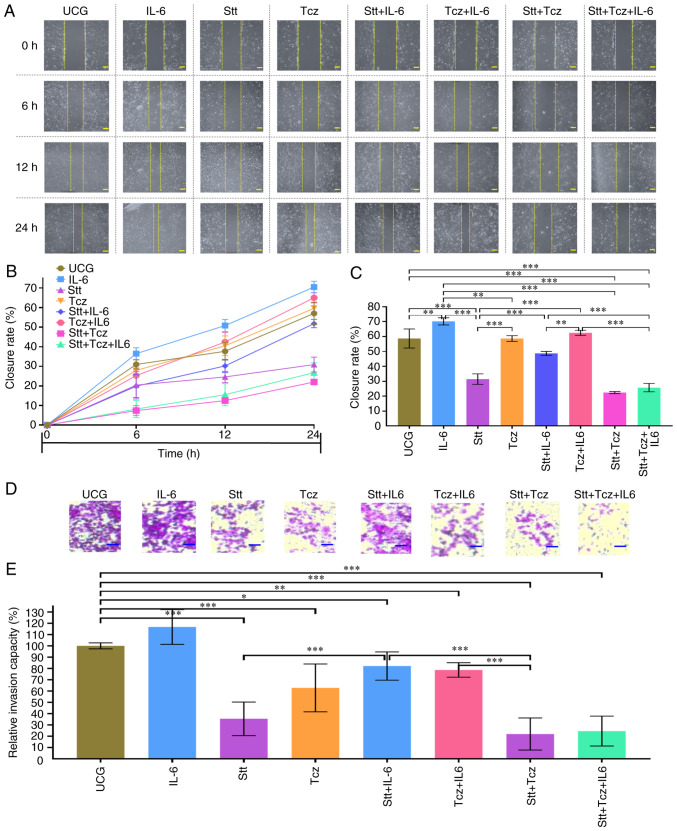
Stt + Tcz treatment decreases migratory and invasive capacity induced by IL-6 in the DU-145 prostate cancer cells. (A) Representative wound-healing assay. Scale bar, 100 µm (photographs were obtained with 40X amplification), (B) closure rate and (C) effect of different treatment at 24 h. (D) Chemotaxis assay was performed to evaluate invasive cells, the scale bars denote 40 µm (100× amplification) and (E) SRB was measured. Data are presented as the mean ± SD of three independent experiments, normalized to respective UCG. *P<0.05, **P<0.01 and ***P<0.001. Stt, stattic; Tcz, tocilizumab; UCG, untreated control group; OD, optical density; SRB, Sulforhodamine B.

To evaluate the effect of the treatment on migration and invasion, cells were treated for 72 h and wound-healing assay was performed (Fig. 7A). IL-6 significantly increased migratory capacity in DU 145 cells (Fig. 7B and C) and Stt decreased cell migration. The effect produced by Stt is partially reversed when IL-6 was added (Stt + IL-6 group) but in the Stt + Tcz group, inhibition of the cell migration capacity was maintained following IL-6 treatment. This demonstrated that Stt + Tcz inhibited migration of DU-145 in the presence or absence of IL-6.

Next, invasive capacity of cells was evaluated via chemiotaxis assay (Fig. 7D and E). There was a similar pattern to that found in the wound-healing assay: IL-6 increased invasive capacity and Stt decreased invasion. Stt + Tcz decreased invasive capacity even when IL-6 was added. This revealed that IL-6 decreased the inhibitory effect produced by Stt alone. However, combined Stt + Tcz increased the inhibitory effect on migratory and the invasive capacity of metastatic DU-145 cells via blockade of the IL-6/IL-6R/STAT-3 axis.

## Discussion

The STAT-3 pathway is involved in metastasis of tumor cells and immunosuppression (28). Therefore, the present study investigated whether blocking IL-6/IL-6R/STAT-3 signaling disrupts proliferation, migration, invasion and clonogenicity of PCa cells; these processes are essential to metastasis in cancer (29).

There were certain differences between cell lines. First, STAT-3 was constitutively phosphorylated in androgen-independent PCa DU-145 cells. Moreover, DU-145 cells exhibited higher migratory and proliferative capacity than 22Rv1 and LNCaP PCa cells. STAT-3 signaling serves a key role in cancer as it is associated with increased proliferative, migratory, invasive and metastatic capacity (30). In particular, in PCa pSTAT-3 and IL-6R are present in 95% of metastatic niches of patients who die of castration-resistant PCa (CRPC); this suggests that in PCa, the activation of STAT-3 mediated by IL-6, IL-10 and EGF serves an important role for tumor progression and development of metastasis (10).

In the present study, PCa cells secreted abundant IL-6 and CXCL8; this secretion was higher in DU-145 cells compared with other cell lines. Furthermore, as reported in other studies, this indicates the key role of IL-6 and IL-8 in autocrine signaling for tumor cells and regulation of the immune response against the tumor (31,32). Previous studies have focused on STAT-3, IL-6R and IL-6 pathways as enhancers of tumor progression. These molecules exert effects through different mechanisms in tumor cells, promoting EMT, proliferation, survival and invasiveness, as well, immune cell infiltration of tumor (neutrophils, natural killer, effector T and dendritic cells), which downmodulates antitumor immunity (32–34). Also contributes to immune suppression via upregulation of inhibitory molecules, such as cytotoxic T lymphocyte-associated protein 4 and PD-L1, and inhibition of M1 and activation of M2 macrophages (28,35–37).

Cancer cells shape their local microenvironment via secretion of soluble molecules to provide necessary conditions that permit tumor growth and proliferation and to prepare them for migration and invasion of other tissue (38). VEGF (39), as well as the cytokines IL-6, IL-8, TGF-β (40–42), facilitate these processes via activation of multiple pathways, including SMAD, NF-κB, WNT and STAT-3 (43,44). These pathways promote EMT via upregulation of transcription factors including Snail family transcriptional repressor 1, zinc finger E-box binding homeobox 1 (Zeb1) and twist family bHLH transcription factor (Twist) (44). These factors downregulate molecules such as E-cadherin and upregulate N-cadherin, vimentin and MMP-2, −3 and −9 secretion, increasing the migratory and invasive capacity of cells (45).

The present results demonstrated that IL-6, CXCL8 and VEGF are expressed in PCa cell lines, predominantly in metastatic DU-145 cells. Increased secretion of IL-6 and VEGF may be associated with metastasis of PCa cells and their aggressive characteristics (4,46). IL-6 serum levels are increased with tumor development in patients with PCa and are higher in patients with metastasis (4). Similarly, high VEGF expression is associated with metastasis and shorter overall survival in patients with PCa (46). VEGF expression is higher in metastatic cells than in the original primary tumor (47). Overexpression of VEGF leads to activation of NF-κB, MAPK and ERK in endothelial cells and increases angiogenesis and lymphangiogenesis via increased cell survival, proliferation, differentiation and migration (48,49).

Although phosphorylation of STAT-3 was observed in non-tumorigenic RWPE-1 cells, this may be mediated by EGF (50). This growth factor is necessary for maintenance and proliferation of RWPE-1 cells in culture (51). Moreover, in DU-145 PCa cells, there was an association between high levels of IL-6, VEGF and IL-6R expression with constitutive activation of STAT-3; this was not observed in other PCa cells (22Rv1 and LNCaP). Furthermore, the present results showed that in DU-145 cells, IL-6 may be involved in activation of STAT-3 in an autocrine manner; IL-6 is higher in tumoral compared with non-tumorigenic tissue, in patients with breast (52) and colorectal cancer (53) and PCa (54,55). This cytokine induces upregulation of vimentin and inhibition of E-cadherin. The expression of these proteins is associated with acquisition of the migratory phenotype of cancer cells (56). IL-6 induces VEGF production in gastric cancer (57). The present results indicated that pSTAT-3 was associated with high vimentin expression and low levels of E-cadherin; DU-145 PCa cells exhibited highest vimentin and lowest E-cadherin expression, leading to high migratory capacity. IL-6R and pSTAT-3 are upregulated in advanced cancer and are detected at higher levels in samples of bone, lymph node and visceral metastasis compared with normal tissue (10). Activated STAT-3 triggers EMT, decreasing E-cadherin and increasing vimentin expression (28), thus inducing proliferation, migration and invasion of cancer cells (58). All of these effects mediated by STAT-3 impact the overall survival of patients with gastric, hepatic and pancreatic cancer, osteosarcoma and PCa (59). Therefore, the present study focused on disrupting the IL-6/STAT-3 pathway in human androgen-independent/highly metastatic PCa DU-145 cells using Stt, an inhibitor of STAT-3, and Tcz, an anti-IL-6R antibody.

The role of STAT-3 in multiple types of disease have been studied to develop a specific treatment to inhibit IL-6, IL-6R, glycoprotein-130, Jak and STAT-3 (15). Here, Stt decreased cell viability at concentrations >5 µM. At concentrations >5 µM, Stt increases cleaved poly (ADP-ribose) polymerase and total and cleaved Caspase-3, as well as the number of apoptotic cells in a model of nasopharyngeal carcinoma (60). However, 10–10,000 ng/ml Tcz did not decrease viability in DU-145 cancer cells. The inhibition of pSTAT-3 produced by Stt + Tcz was higher than Stt or Tcz alone. In DU-145 cells, the addition of IL-6 decreased the inhibitory effect of Stt or Tcz alone, however, combination of Stt + Tcz sustained this inhibitory effect despite high concentrations of IL-6.

Although IL-6 is a key inducer of STAT-3 activation, blocking IL-6R using Tcz alone is insufficient to abolish the effects of IL-6, indicating the involvement of other pathways in STAT-3 activation (61). STAT-3 is activated by cytokines (IL-6, IL-2, IL-10 and IL-2), growth factors (EGF and granulocyte-macrophage colony-stimulating factor) (62), and the formation of complexes including integrin-EGFR and integrin-protein tyrosine kinase 2-JAK (63). However, the present study demonstrated that combination of Tcz and Stt enhanced the inhibitory effect on STAT-3 phosphorylation. The present results indicated that combination of Stt and Tcz disrupted the IL-6R/STAT-3 pathway. IL-6 increased VEGF secretion; this was reversed when cells were treated with Stt + Tcz. In the present study, the same effect was observed for vimentin: IL-6 induced its expression and Stt + Tcz decreased expression, while the opposite effect was observed for E-cadherin. STAT-3 induces upregulation of vimentin and downregulation of E-cadherin in colorectal cell lines LoVo, and SW1116 (14), which facilitates EMT for successful metastasis (14,45,64). Treatment with Stt decreased proliferative capacity of PCa cells. This effect was higher when DU-145 cells were treated with Stt + Tcz in a time-dependent manner, also inhibit the clonogenic capacity.

The same effect has been observed with Stt in BTIC glioblastoma cells (65); activation of STAT-3 induces upregulation of c-Myc, Cyclin D1 and Bcl-xL and protects against apoptosis to promote cell survival and proliferation (66). Other studies have shown that Stt and Napabucasin (a small molecule that targets STAT-3) decrease cell renewal potential and the number of colonies, thus preventing metastasis into bone tissue of PCa in a mouse model (67) also, Terphenylin suppress the tumor growth and metastasis in mouse model of gastric cancer (68) In addition, STAT-3 inhibition via galiellalactone decreases formation of metastatic, clonogenic and spherical stem PCa and gastric cancer cells (69,70). Androgen deprivation contributes to development of CRPC in patients (71). The activation of STAT-3 induced by IL-6 increase the resistance of LNCaP cells to Enzalutamide (a second-generation androgen antagonist) by re-activation of androgen receptor (AR) and nuclear translocation (72). Treatment with AR and STAT-3 inhibitors decreases the effect on PCa cell proliferation induced by AR activation via the IL-6 pathway in a Dihydrotestosterone-independent manner (72,73). Therefore, IL-6/STAT-3 pathway blockade increases response to AR inhibitors (74).

**Figure 8.**
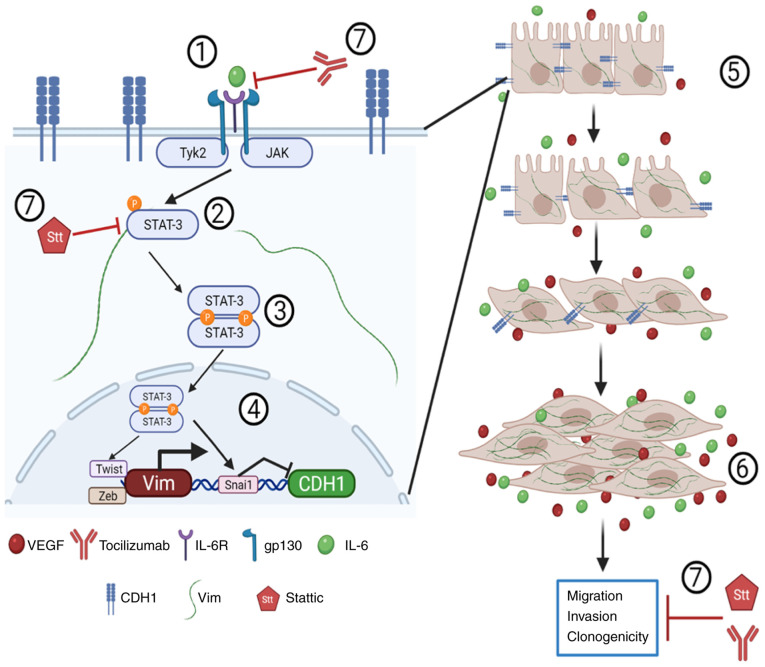
Stt + Tcz increases expression of E-cadherin and decrease Vim expression and the proliferative, migratory, clonogenic, and invasive capacity. The interaction between IL-6 and IL-6R (1) activates JAK kinases and phosphorylates STAT-3 (2), while pSTAT-3 achieves dimerization (3). The STAT-3 dimer moves into the nucleus and upregulates transcription factors (Twist, Zeb, Snai1) that increase vimentin and VEGF and decrease E-cadherin expression (4). This induces morphology changes (5), as well as increasing migratory, invasive and clonogenic capacity in the tumor cells (6). Stt bind to STAT-3 SH2 domain, inhibiting the phosphorylation, and Tcz block interaction of IL-6/IL-6R. Stt + Tcz increase E-cadherin and decreases VEGF and Vim expression, and also the proliferative, clonogenic, migratory and invasive capacity of the DU-145 metastatic cells (7). CDH1, E-cadherin; Vim, vimentin; R, receptor; JAK, Janus kinase; p, phosphorylated; Stt, stattic; Snai1, snail family transcriptional repressor 1; Zeb1, zinc finger E-box binding homeobox 1; Tyk2, tyrosine kinase 2; E-, epithelial. Created with BioRender.com.

Migration and invasion are important in cancer cells for development of metastasis. IL-6 increases migration and invasion by upregulating molecules such as vimentin, MMP-2 and MMP-9 and downregulating E-cadherin (14,75). Decreased expression of vimentin and the upregulation of E-cadherin molecules produced by Stt and Tcz alone or in combination contributes to decreased migratory and invasive capacity in DU-145 PCa cells (Fig. 8). Finally, the present results revealed that combined Stt + Tcz decreased the migratory and invasive capacity of cells even following addition of IL-6.

STAT-3 has key implications in cancer, cytokine storm, autoimmune disease and infection, included SARS-CoV2 (2), the development of therapies such as Stt + Tcz could improve the actual treatments again this pathway The present study demonstrated that Stt + Tcz was more effective than Stt or Tcz alone at inhibiting the IL-6/IL-6R/STAT-3 axis. Stt + Tcz decreased proliferative, clonogenic, migratory and invasive ability of DU-145 PCa cells by regulating protein levels of VEGF, vimentin, and E-cadherin. Disruption of the IL-6/IL-6R/STAT-3 axis using Stt and Tcz may be a therapeutic target in PCa based on the molecular characteristics of the tumor cells.

## Supplementary Material

Supporting Data

## Figures and Tables

**Table I.** Reverse transcription-quantitative PCR analysis of epithelial-mesenchymal transition-associated genes.

Gene	Primer sequence, 5′→3′	Annealing temperature, °C	Extension time, sec
GRHL2	F: GAAGAAGGGACAAAGCGAGTG	56	12
	R: CCAAACCCAGGGCTAGATTTC		
CDH1	F: CCTCATGAGTGTCCCCCGGTAT	60	11
	R: CTCGCCGCCTCCGTACATGTC		
FZD7	F: GGCCTGCCTGCTAGAATCCTAA	56	14
	R: GATTTGCGCTGCTTTGCCTAT		
JAG1	F: TGCGAACATCACATTTACCTT	52	9
	R: CAATCATCCCGTATATCTT		
ERBB3	F: CCATTCCCAGCGCCACA	58	9
	R: GTGCCTTCTCCTCCGGTTCAT		
CTNNB1	F: AGCTTCCAGACACGCTATCAT	56	15
	R: ATTTGAAGGCAGTCTGTCGTAA		
SNAI1	F: CGAAAGGCCTTCAACTGCAA	62	11
	R: CGCCTGGCACTGGTACTTCTT		
ZEB1	F: CGGAAGACAGAAAATGGAA	62	14
	R: CTTCCGCTTCTCTCTTAGAGT		
VIM	F: TGCCGTTGAAGCTGCTAACTA	58	15
	R: CGTGATGCTGAGAAGTTTCGT		
RPL32	F: GCATTGACAACAGGGTTCGTA	62	14
	R: ATTTAAACAGAAAACGTGCACA		

F, forward; R, reverse; GRHL2, grainyhead-like transcription factor 2; CDH1 epithelial cadherin; FZD7, frizzled class receptor 7; JAG1, jagged canonical Notch ligand 1; Erbb3, Erb-b2 receptor tyrosine kinase 3; Snail1, snail family transcriptional repressor 1; Rpl32, ribosomal protein L32; ZEB1, Zinc finger E-box-binding homeobox 1.

## Data Availability

The datasets used and/or analyzed during the current study are available from the corresponding author on reasonable request.

## Abbreviations

AR: androgen receptor
CRPC: castration-resistant prostate cancer
EGF: epidermal growth factor
G-CSF: granulocyte colony stimulating factor
PCa: prostate cancer
pSTAT-3: phosphorylated signal transducer and activator of transcription 3
Stt: stattic
Tcz: tocilizumab
TGF: transforming growth factor
UCG: untreated control group
