# Supplementary material for: Dual STAT-3 and IL-6R inhibition with stattic and tocilizumab decreases migration, invasion and proliferation of prostate cancer cells by targeting the IL-6/IL-6R/STAT-3 axis
Source: Oncol Rep. 2022 Jun 14;48(2):138. doi: 10.3892/or.2022.8349 (PMC9245073; doi:10.3892/or.2022.8349)

Figure S1. Expression of cytokines secreted by the cell lines RWPE-1, 22Rv1, LNCaP and DU-145. Data show as concentration pg/ml.

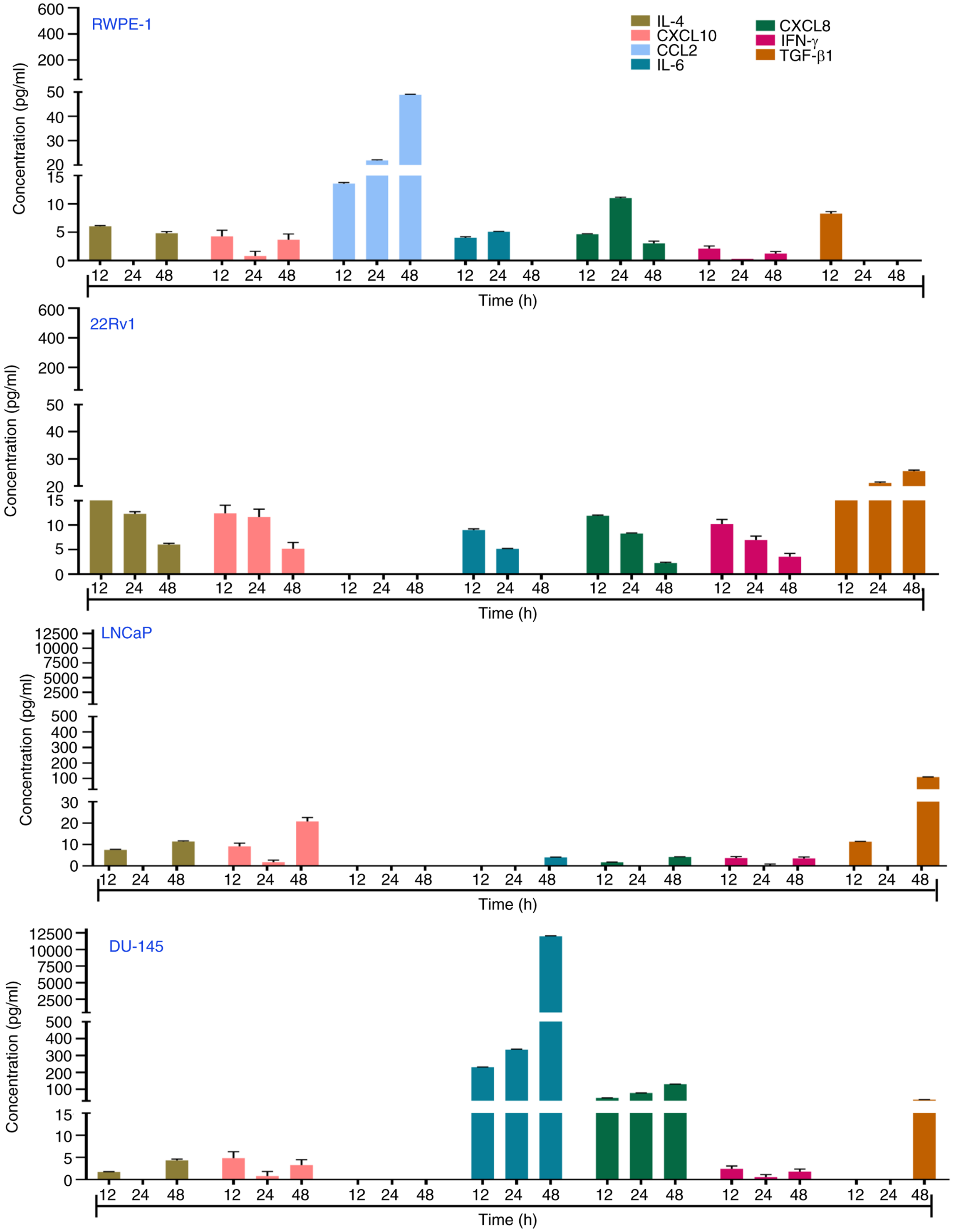

Figure S2. Expression of growth factors secreted by the cell lines RWPE-1, 22Rv1, LNCaP and DU-145. Data show as concentration pg/ml.

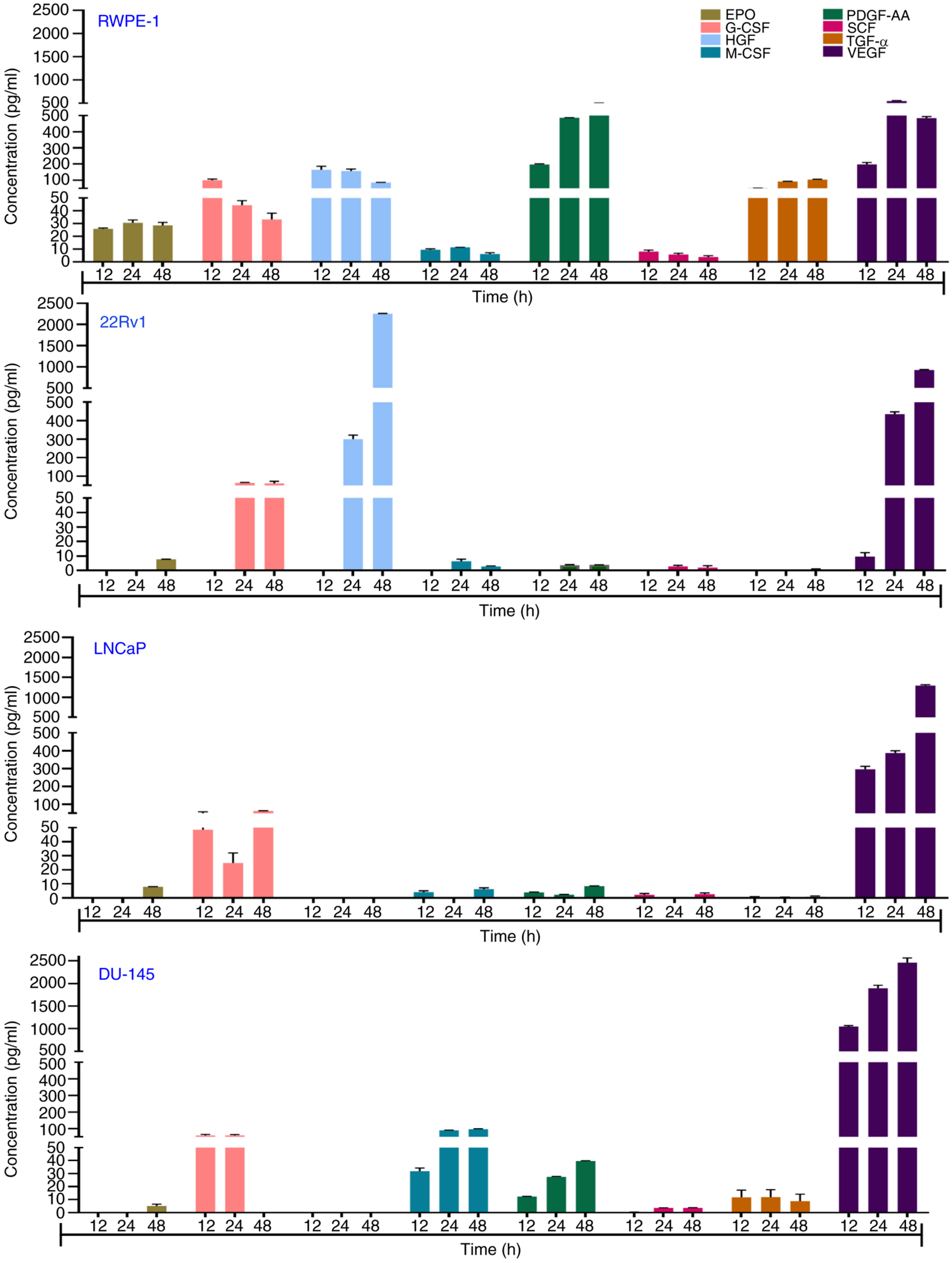

Supplement: Supporting Data [file Supplementary_Data.pdf]
